# Low T3 syndrome is associated with 30-day mortality in adult patients with fulminant myocarditis

**DOI:** 10.3389/fendo.2023.1164444

**Published:** 2023-05-31

**Authors:** Guangrui Miao, Shuo Pang, Yuanhang Zhou, Mingxuan Duan, Linpeng Bai, Xiaoyan Zhao

**Affiliations:** Department of Cardiology, The First Affiliated Hospital of Zhengzhou University, Zhengzhou, China

**Keywords:** low T3 syndrome, fulminant myocarditis, 30-day mortality, biomarkers, prognosis

## Abstract

**Background:**

Fulminant myocarditis (FM) is a critical disease with high early mortality. Low triiodothyronine syndrome (LT3S) was a strong predictor of poor prognosis of critical diseases. This study investigated whether LT3S was associated with 30-day mortality in FM patients.

**Methods:**

Ninety-six FM patients were divided into LT3S (n=39, 40%) and normal free triiodothyronine (FT3) (n=57, 60%) groups based on serum FT3 level. Univariable and multivariable logistic regression analyses were performed to identify independent predictors of 30-day mortality. Kaplan–Meier curve was used to compare 30-day mortality between two groups. Receiver operating characteristic (ROC) curve and decision curve analysis (DCA) were used to assess the value of FT3 level for 30-day mortality prediction.

**Results:**

Compared to normal FT3 group, LT3S group had higher incidence of ventricular arrhythmias, worse hemodynamics, worse cardiac function, more severe kidney impairment, and higher 30-day mortality (48.7% vs. 12.3%, P<0.001). In univariable analysis, LT3S (odds ratio [OR]:6.786, 95% confidence interval [CI]:2.472-18.629, P<0.001) and serum FT3 (OR:0.272, 95%CI:0.139-0.532, P<0.001) were significant strong predictors of 30-day mortality. After adjustment for confounders in multivariable analysis, LT3S (OR:3.409, 95%CI:1.019-11.413, P=0.047) and serum FT3 (OR:0.408, 95%CI:0.199-0.837, P=0.014) remained independent 30-day mortality predictors. The area under the ROC curve of FT3 level was 0.774 (cut-off: 3.58, sensitivity: 88.46%, specificity: 62.86%). In DCA, FT3 level showed good clinical-application value for 30-day mortality prediction.

**Conclusion:**

In FM patients, LT3S could independently predict 30-day mortality. FT3 level was a strong 30-day mortality predictor and a potentially useful risk-stratification biomarker.

## 1 Introduction

Acute myocarditis is defined as impaired cardiac function as a result of myocardial inflammatory injuries of various causes, and characterized by reduced systolic function and cardiac arrhythmias (1–3). Fulminant myocarditis (FM) is the most serious and specific type of acute myocarditis, which often has a rapid progression and manifests with hemodynamic collapse, followed by refractory cardiogenic shock, life-threatening ventricular arrhythmias or electrical storms, and other complications (4, 5). While FM had a high early mortality, this condition had a good long-term prognosis (6–9). Therefore, it is very important to find biomarkers for accurate prediction of FM prognosis so that intervention measures could be provided as early as possible.

Low triiodothyronine (T3) syndrome (LT3S), also known as euthyroid sick syndrome, is characterized by reduced serum T3 levels and normal thyroid-stimulating hormone (TSH) concentrations (10–12). LT3S was considered an adaptative response to preserve energy in acute and chronic severe diseases (13). Accumulating evidence showed that LT3S was strongly associated with poor prognosis in critical illnesses (14, 15). In the past few years, the interplay between LT3S and cardiovascular disease has attracted great attention (16–18). However, whether LT3S was associated with poor prognosis of FM patients was uncertain. Therefore, this study investigated whether LT3S was associated with 30-day mortality of FM patients.

## 2 Materials and methods

### 2.1 Study design and participants

**Figure 1:**
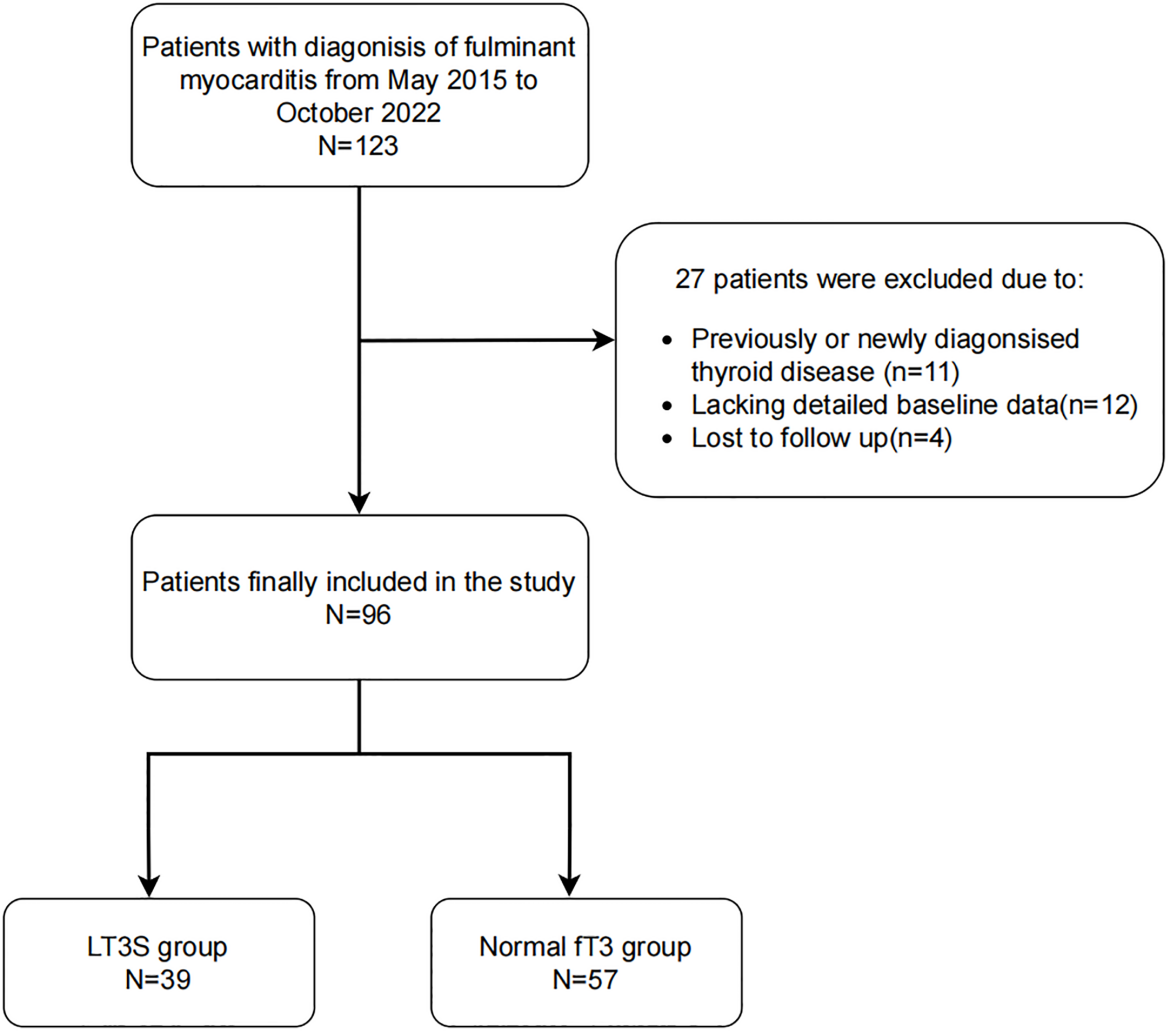
The flow chart of the enrollment of 96 patients with FM.

In this single-center, retrospective study, 123 FM patients (age≥18 years) were enrolled in the First Affiliated Hospital of Zhengzhou University (Zhengzhou, China) from May 2015 to October 2022. According to the recommendations in the Chinese Expert Consensus (19), FM is defined as acute myocarditis with hemodynamic instability requiring high-dose vasopressors (≥5 µg/[kg·min] of dopamine, dobutamine or other inotropic equivalents) or mechanical circulatory support (MCS), despite maximal medical treatment. Eleven patients were excluded because of previous or new diagnoses of thyroid diseases. Additionally, due to the lack of detailed baseline data and loss to follow-up, 12 and 4 patients were excluded, respectively. The study endpoint was 30-day mortality, obtained by a trained doctor through reviewing patient’s hospital record and interviewing the patients by phone. The flow chart of the enrollment process of 96 FM patients was showed in **Figure 1**.

The study was conducted in compliance with the requirement for medical research ethics in the Declaration of Helsinki (2013 revision). Our study was approved by the Ethics Committee of the First Affiliated Hospital of Zhengzhou University (ID: 2022-KY-1446). In addition, all the candidates signed the informed consent before we conducted the study.

### 2.2 Data collection

We collected patients’ clinical characteristics, including their demographic data (gender and age), comorbidities (hypertension, diabetes mellitus, and dyslipidemia), symptoms (fever, palpitations, chest pain, dyspnea, and syncope), electrocardiogram findings at admission (ST-T change, complete atrioventricular block, bundle branch block, supraventricular tachycardia, and ventricular tachycardia or ventricular fibrillation), vital signs at admission (systolic pressure, diastolic pressure, and heart rate), laboratory results (free T3 [FT3], free thyroxine [FT4], TSH, creatine, aspartate transaminase, alanine aminotransferase, albumin, C-reactive protein [CRP], peak N-terminal pro-B-type natriuretic peptide [NT-proBNP], and peak Troponin I [TnI]), left ventricular ejection fraction (LVEF), medications (anti-viral therapy, glucocorticoid therapy, and intravenous immunoglobulin), and life support treatment (MCS, including intra-aortic balloons pump and extracorporeal membrane oxygenation, temporary pacing, and continuous renal replacement therapy [CRRT]) from electronic medical information recording system.

### 2.3 Thyroid function test

Serum FT3, FT4, and TSH levels were measured on the second day of admission by using chemoluminescence immunoassay (UniCel Dxl 800 Access, Beckman Coulter, Brea, CA, USA) in the Department of Nuclear Medicine of the First Affiliated Hospital of Zhengzhou University. The normal reference values for thyroid function tests are as follows: FT3: 3.28-6.47 pmol/L, FT4: 7.9-18.4 pmol/L, and TSH: 0.56-5.91 µIU/mL. LT3S was defined as FT3<3.28 pmol/L with normal TSH level, with or without FT4<7.9 pmol/L.

### 2.4 Statistical analysis

All statistical analyses were carried out using SPSS statistics software (version 24.0) and R software (version 4.2.2). First, we used Kolmogorov–Smirnov test to verify the normality of the distribution of continuous variables. Variables with normal distributions were presented as mean ± standard deviation (SD) and compared between two groups by Student’s t test, while non-normally distributed variables were expressed as medians with interquartile ranges (IQR) and compared between two groups by Mann–Whitney U test. Categorical variables were presented as counts (percentages) and compared by the χ2 or Fisher’s exact test. Second, univariable logistic regression analysis was performed to identify variables associated with 30-day mortality. All significant variables in the univariable analysis were included in the multivariable analysis. We used Kaplan–Meier curve to describe and compare 30-day mortality between the groups. Third, the receiver operating characteristic (ROC) curve was performed and the area under the ROC curve (AUC) was calculated to assess the predictive ability of FT3 level for 30-day mortality. Decision curve analysis (DCA) was used to assess the clinical value of FT3. All tests were 2-sided, and P<0.05 was considered statistically significant.

## 3 Results

### 3.1 Clinical characteristics

Ninety-six participants (male proportion: 42.7%) were included in this study, with a mean age of 42 ± 14 years. Clinical characteristics of FM patients were shown in **Table 1**. Patients were divided into LT3S (n=39, 40%) and normal FT3 (n=57, 60%) groups. No remarkable difference was observed in demographics and comorbidities between two groups. A higher proportion of patients with syncope and ventricular tachycardia/ventricular fibrillation was observed in LT3S group than in normal FT3 group. Compared to normal FT3 group, LT3S patients had lower systolic blood pressure and faster heart rate. Additionally, serum FT3, FT4, TSH levels and LVEF were significantly lower in LT3S group than in normal FT3 group. Serum creatine, CRP, peak NT-proBNP, and peak TnI levels were higher in LT3S group than in normal FT3 group. For the treatment of FM, more patients in LT3S group accepted MCS, ventilator, and CRRT as compared to those in normal FT3 group.

**Table 1:** Clinical characteristics of the patients with FM.

Variable	Total(n=96)	LT3S group (n=39, 40%)	Normal FT3 group (n=57, 60%)	*t/Z/χ^2^*	*P* Value
Demographics					
Male, n (%)	41 (42.7)	18 (46.2)	23 (40.4)	0.319	0.572
Age, years	42 ± 14	44 ± 15	40 ± 14	1.409	0.162
Comorbidities					
Hypertension, n (%)	12 (12.5)	6 (15.4)	6 (10.5)	0.154	0.695
Diabetes, n (%)	7 (7.3)	4 (10.3)	3 (5.3)	0.275	0.6
Dyslipidemia, n (%)	6 (6.3)	3 (7.7)	3 (5.3)	0.003	0.957
Symptoms					
Fever, n (%)	43 (44.8)	19 (48.7)	24 (42.1)	0.409	0.522
Palpitations, n (%)	30 (31.3)	13 (33.3)	17 (29.8)	0.133	0.716
Chest pain, n (%)	34 (35.4)	17 (43.6)	17 (29.8)	1.918	0.166
Dyspnea, n (%)	43 (44.8)	21 (53.8)	22 (38.6)	2.178	0.14
Syncope, n (%)	22 (22.9)	13 (33.3)	9 (15.8)	4.035	0.045
ECG findings at admission					
ST-T change, n (%)	38 (39.6)	19 (48.7)	19 (33.3)	2.292	0.13
Complete AVB, n (%)	26 (27.1)	12 (30.8)	14 (24.6)	0.452	0.501
Bundle branch block, n (%)	44 (45.8)	18 (46.2)	26 (45.6)	0.003	0.958
Supraventricular tachycardia, n (%)	10 (10.4)	6 (15.4)	4 (7.0)	0.956	0.328
VT/VF, n (%)	21 (21.9)	13 (33.3)	8 (14.0)	5.046	0.025
Vital Signs at admission					
SBP, mmHg	95 ± 12	92 ± 14	98 ± 10	-2.192	0.032
DBP, mmHg	66 ± 12	63 ± 13	67 ± 10	-1.475	0.145
HR, beats/min	99 (78, 121)	108 (84, 131)	91 (72, 111)	-2.149	0.032
Laboratory tests					
FT3, pmol/L	3.57 (2.86, 4.32)	2.78 (2.34, 2.98)	4.17 (3.72, 4.60)	-8.207	<0.001
FT4, pmol/L	12.09 (9.93, 13.86)	11.78 (8.87, 13.19)	12.39 (10.28, 14.74)	-2.354	0.019
TSH, µIU/ml	1.94 (1.03, 3.02)	1.26 (0.76, 2.11)	2.20 (1.34, 3.43)	-3.577	<0.001
Creatine, μmoI/L	81 (59, 111)	109 (83, 154)	68 (55, 87)	-5.085	<0.001
AST, U/L	169 (70, 408)	284 (77, 563)	136 (66, 235)	-1.928	0.054
ALT, U/L	80 (43, 318)	120 (46, 421)	69 (37, 195)	-1.488	0.137
Albumin, g/L	35.7 (31.5, 39.1)	34.4 (30, 40.0)	36.1 (32.4, 38.3)	-0.836	0.403
CRP, mg/L	41.3 (16.6, 81.2)	52.8 (20.3, 100.0)	35.0 (10.0, 66.9)	-2.044	0.041
Peak NT-proBNP, ng/L	9938 (4753, 16661)	15000 (8516, 26489)	8501 (3617, 13465)	-3.772	<0.001
Peak TnI, μg/L	4.7 (1.8, 15.2)	7.32 (3.23, 20.52)	3.66 (1.22, 10.68)	-2.328	0.02
LVEF at admission, %	42 (34, 45)	37 (33, 43)	43 (36, 47)	-2.64	0.008
Medications					
Anti-viral therapy, n (%)	81 (84.4)	34 (87.2)	47 (82.5)	0.392	0.531
Glucocorticoid therapy, n (%)	74 (77.1)	31 (79.5)	43 (75.4)	0.215	0.643
IVIG, n (%)	59 (61.5)	27 (69.2)	32 (56.1)	1.675	0.196
Life support treatment					
MCS, n (%)	46 (47.9)	26 (66.7)	20 (35.1)	9.253	0.002
Temporary pacing, n (%)	20 (20.8)	9 (23.1)	11 (19.3)	0.2	0.654
CRRT, n (%)	25 (26.0)	16 (41.0)	9 (15.8)	7.657	0.006

LT3S, low triiodothyronine syndrome; ECG, electrocardiogram; AVB, atrioventricular block; VT, ventricular tachycardia; VF, ventricular fibrillation; SBP, systolic blood pressure; DBP, diastolic blood pressure; HR, heart rate; FT3, free triiodothyronine; FT4, free thyroxine; TSH, thyroid-stimulating hormone; AST, aspartate transaminase; ALT, alanine aminotransferase; CRP, C-reactive protein; NT-proBNP, N-terminal pro-B-type natriuretic peptide; TnI, Troponin I; LVEF, left ventricular ejection fraction; IVIG, intravenous immunoglobulin; MCS, mechanic circulatory support; CRRT, continuous renal replacement therapy.

### 3.2 Association between LT3S and 30-day mortality

Univariable and multivariable logistic analyses were performed, with results shown in **Table 2**. In univariable analysis, LT3S (odds ratio [OR]:6.786, 95% confidence interval [CI]:2.472-18.629, P<0.001) and serum FT3 (OR:0.272, 95%CI:0.139-0.532, P<0.001) were independent predictors of 30-day mortality. LT3S (OR:3.409, 95%CI:1.019-11.413, P=0.047) was a strong predictor for 30-day mortality in Model 1. Additionally, serum FT3 (OR:0.408, 95%CI:0.199-0.837, P=0.014) was correlated with 30-day mortality after adjusting for confounding variables in Model 2.

**Table 2:** Logistic regression for 30-day mortality of FM.

Variables	Univariable analysis		Model1		Model2		
	OR (95%CI)	*P* Value	Adjusted OR (95%CI)	*P* Value	Adjusted OR (95%CI)	*P* Value	
LT3S	6.786(2.472, 18.629)	<0.001	3.409(1.019, 11.413)	0.047		
FT3	0.272(0.139, 0.532)	<0.001			0.408(0.199, 0.837)	0.014
Age	1.022(0.991, 1.055)	0.166				
Gender, male	0.433(0.173, 1.084)	0.074				
VT/VF	3.352(1.210, 9.288)	0.020	1.442(0.397, 5.233)	0.578	1.573(0.429, 5.771)	0.495
SBP	0.956(0.920, 0.994)	0.022	0.964(0.917, 1.013)	0.144	0.968(0.921, 1.017)	0.198
HR	1.016(1.001, 1.031)	0.033	1.012(0.995, 1.030)	0.179	1.011(0.993, 1.029)	0.233
Creatine	1.008(0.999, 1.016)	0.057				
Peak NT-proBNP*	1.065(1.014, 1.119)	0.012	0.993(0.929, 1.062)	0.838	0.999(0.936, 1.067)	0.987
Peak TnI	1.038(1.007, 1.070)	0.015	1.023(0.986, 1.061)	0.231	1.022(0.984, 1.060)	0.263
LVEF at admission	0.902(0.840, 0.968)	0.004	0.931(0.851, 1.018)	0.117	0.931(0.850, 1.021)	0.127
CRRT	2.933(1.108, 7.768)	0.030	1.732(0.460, 6.521)	0.417	1.392(0.348, 5.561)	0.640

LT3S, low triiodothyronine syndrome; FT3, free triiodothyronine; VT, ventricular tachycardia; VF, ventricular fibrillation; SBP, systolic blood pressure; HR, heart rate; NT-proBNP, N-terminal pro-B-type natriuretic peptide; TnI, Troponin I; LVEF, left ventricular ejection fraction; CRRT, continuous renal replacement therapy.

Model1 includes LT3S and all significant variable at the univariate analysis. FT3 and all significant variable at the univariate analysis were included in Model 2.

* per 1000ng/L increase.

**Figure 2:**
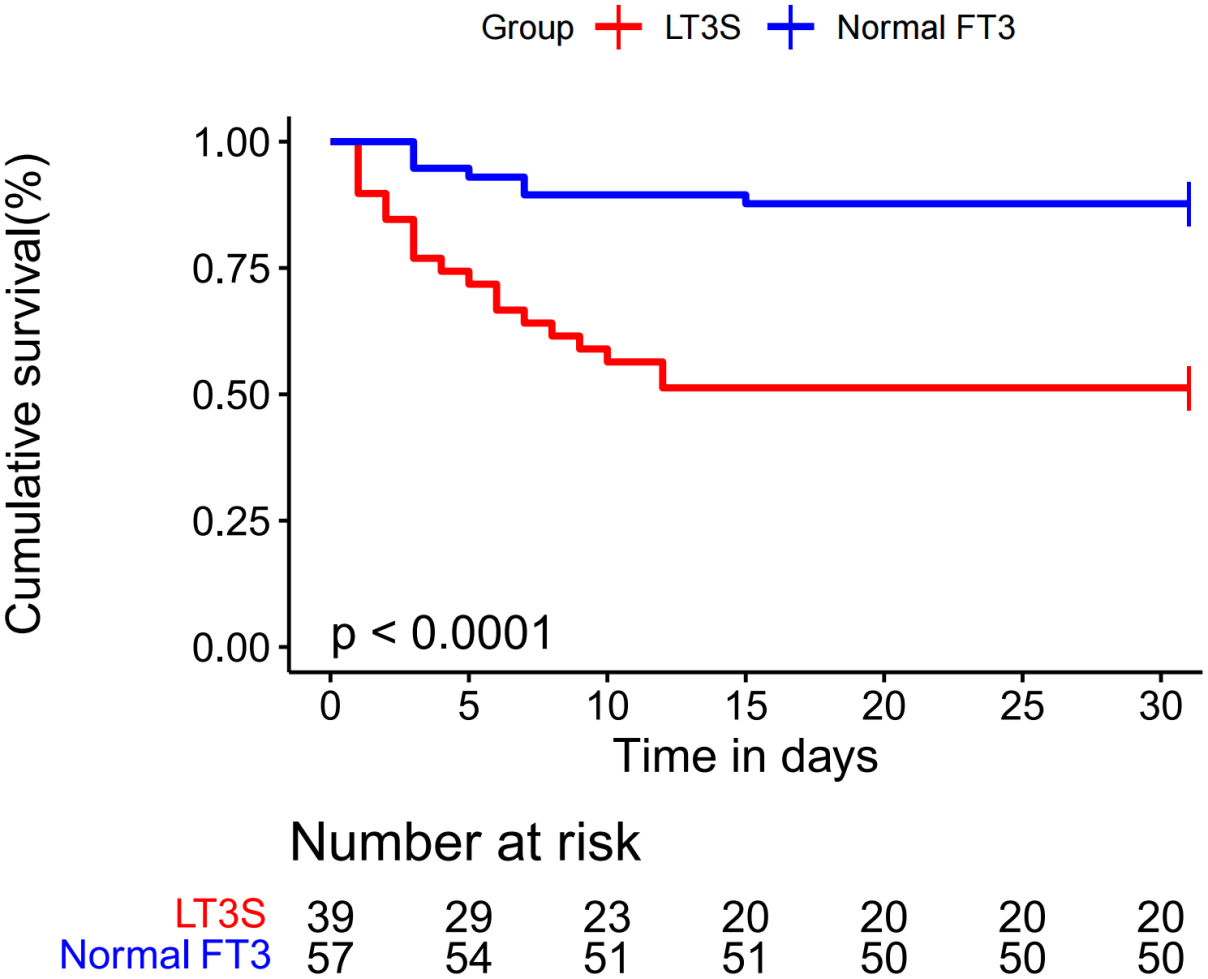
Kaplan–Meier curve for 30-day mortality in LT3S group versus in normal FT3 group.

The 30-day survival curve of FM patients was displayed in **Figure 2**. LT3S patients had a higher 30-day mortality than patients in normal FT3 group (48.7% *vs.* 12.3%, log-rank P<0.001).

### 3.3 Predictive value of FT3 level for30-day mortality

The comparisons of AUCs and FT3 cut-off values for 30-day mortality were presented in **Table 3**. The overall AUC and FT3 cut-off value for 30-day mortality were 0.774 and 3.58, respectively. The overall sensitivity and specificity of FT3 level for 30-day mortality prediction were 88.46% and 62.86%, respectively. Additionally, in subgroup analysis, the predictive power of FT3 level for 30-day mortality was not affected by gender (male *vs.* female: 0.767 *vs.* 0.809), age (≥50 years *vs.* <50 years: 0.724 *vs.* 0.786), LVEF (≥40% *vs.* <40%: 0.765 vs. 0.778), CRRT (yes *vs.* no: 0.854 *vs.* 0.709), and MCS (yes *vs.* no: 0.756 *vs.* 0.779). Details of the cut-off values, sensitivity, and specificity of different subgroups were also presented in **Table 3**.

**Table 3:** Area under the receiver operating characteristic curve of the value of FT3 for predicting 30-day mortality.

	Total (N=96)	AUC (95%CI)	Cut-off value	Sensitivity (%)	Specificity (%)
Overall patients	96	0.774(0.678, 0.854)	3.58	88.46	62.86
Gender					
Male	41	0.767(0.609, 0.884)	3.58	86.67	65.38
Female	55	0.809(0.680, 0.902)	3.43	90.91	68.18
Age					
≥50 years	31	0.724(0.535, 0.868)	3.52	90.00	52.38
<50 years	65	0.786(0.666, 0.878)	3.12	81.25	77.55
LVEF at admission					
≥40%	54	0.765(0.630, 0.870)	3.01	77.78	80.00
<40%	42	0.778(0.623, 0.891)	3.58	88.24	64.00
MCS					
Yes	46	0.756(0.607, 0.870)	2.72	53.33	87.10
No	50	0.779(0.639, 0.884)	3.58	81.82	82.05

LVEF, left ventricular ejection fraction; MCS, mechanic circulatory support.

**Figure 3:**
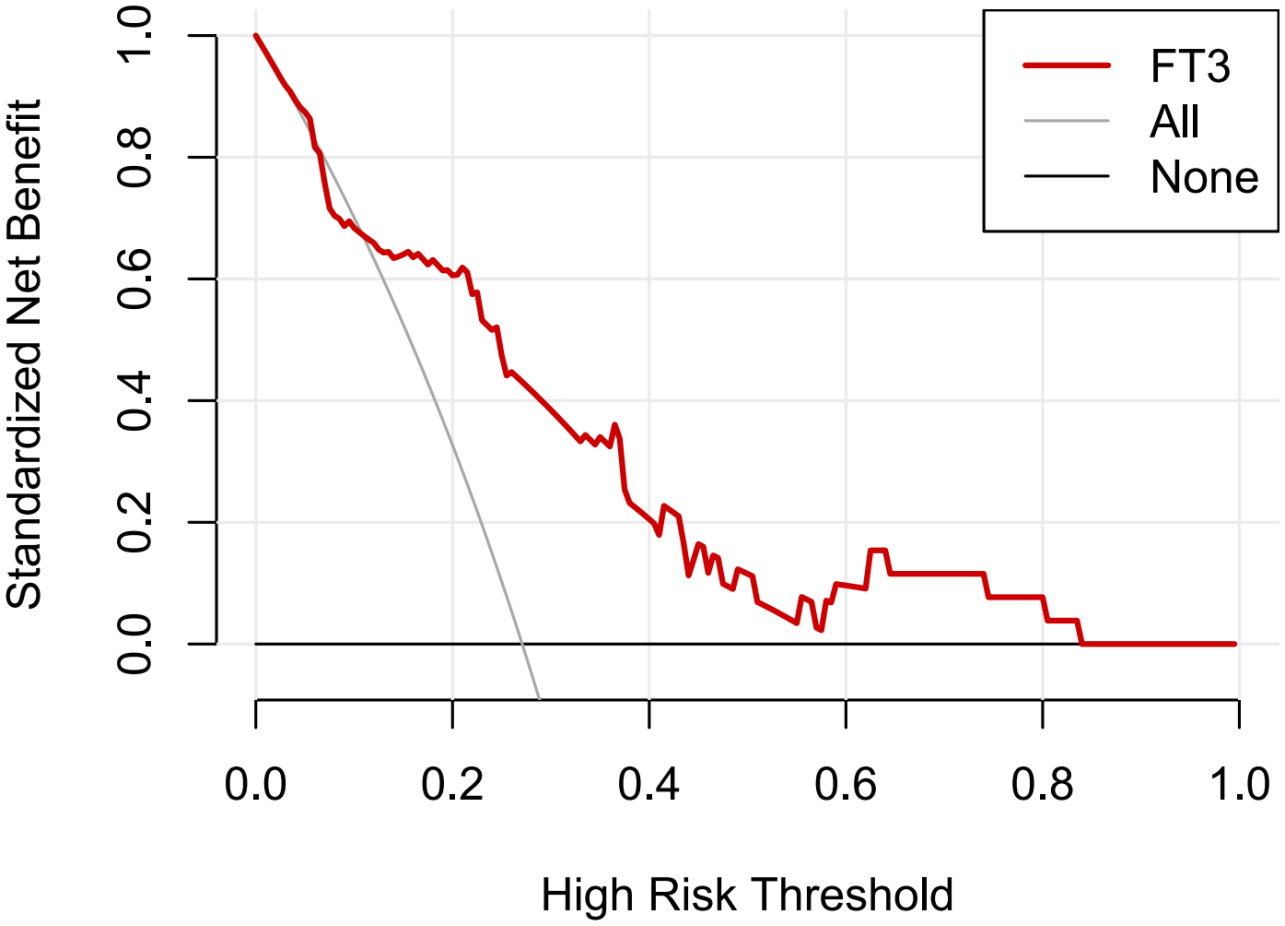
Decision curve analysis (DCA) of FT3 levels in predicting 30-day mortality. The y-axis was used to test the net benefit. The gray line indicated that all patients died within 30 days, while the black line indicated that all patients survived for over 30 days. The red line displayed the benefit of FT3 level for 30-day mortality.

Furthermore, the DCA curves (**Figure 3**) suggested that if the threshold probability is 0.15-0.80, using the FT3 level to predict 30-day mortality in our study could add more benefit, which indicates FT3 level had good clinical-application value for predicting 30-day mortality.

## 4 Discussion

In this study, by collecting and analyzing clinical characteristics of 96 FM patients, we found that patients with LT3S had a higher proportion of ventricular arrhythmias, worse hemodynamics, worse cardiac systolic function, more severe kidney impairment. In multivariate analysis, after adjusting for potential confounders, we identified LT3S and FT3 level as significant independent predictors of 30-day mortality, which provided more risk-stratification information for FM patients. Additionally, FT3 level moderately predicted 30-day mortality regardless of gender, age, LVEF level, and MCS treatment.

In cardiovascular disease, LT3S had been extensively studied. Nevertheless, no studies have reported the incidence of LT3S in FM patients until now. According to our study, LT3S occurred in 40% of FM patients. This LT3S prevalence in FM is higher than those reported for other cardiovascular diseases (16, 20, 21), and might be explained by the FM treatment, which often included drugs, such as dopamine, glucocorticoids, amiodarone, and others, that could affect thyroid function measurement. Additionally, renal dysfunction and severe inflammatory process, often accompanied with FM, also induced LT3S. Meanwhile, the 30-day mortality in LT3S group was 48.7%, which was significantly higher than that of normal FT3 group. A systematic review and meta-analysis of 41 studies showed that LT3S was an independent predictor for all-cause mortality, cardiac mortality, and MACE in heart diseases (22). Su et al. revealed that the patients with LT3S had higher in-hospital cardiovascular mortality in patients with acute myocardial infarction (23). Recently, in the study of Zhao et al., LT3S could predict poor prognosis in adult patients with acute myocarditis (24). Collectively, accumulating evidence showed that LT3S was associated with poor prognosis in cardiovascular disease.

However, the underlying mechanisms of the association between LT3S and FM remain uncertain. Several observations could explain the relationship between LT3S and FM. First, consistent with previous report, our LT3S patients had worse cardiac function and hemodynamics (25). Thyroid hormones played a pivotal role in the cardiovascular system, which regulated cardiovascular hemodynamics, cardiac filling, and systolic contractility (26). Particularly, T3 can decrease peripheral vascular resistance and increase heart rate as well as left ventricular myocardial contractility (27, 28). Therefore, low T3 levels will reduce cardiac function and induce structural changes of the myocardium. Second, we noted that LT3S patients had more severe myocardial injury, which might further aggravate FM progression. To date, no studies provided an explanation for the association between myocardial injury and LT3S in FM patients. Nevertheless, some studies showed that thyroid hormones had a significant cardioprotective effect in patients with acute myocardial infarction, which could reduce myocardial injury and reverse left ventricular remodeling by activating cytoprotection, stimulating cell growth, and triggering neovascularization and metabolic adaptation (29, 30). Alexandre et al. showed various protective functions of thyroid hormones in the ischemia/reperfusion rat model (31). As such, the mechanism of how LT3S affects the myocardial injury requires further investigation. Third, there is a crosstalk between LT3S and inflammatory processes. Thyroid hormones can modulate the inflammatory and immune responses by genomic and non-genomic mechanisms (32). For example, thyroid hormones can regulate pro-inflammatory macrophage responses by enhancing phagocytic activity and promoting nitric oxide production in macrophage cell lines (33). Besides, Ma et al. reported that subclinical hypothyroidism was positively correlated with reactive oxygen species generation via nicotinamide adenine dinucleotide phosphate oxidase activation, suggesting an inhibitory thyroid hormone effect on neutrophil function (34). Therefore, it is plausible that impaired thyroid hormone function results in the pathogenesis of inflammation processes. Moreover, various inflammatory cytokines play a critical role in the pathogenesis of LT3S (35, 36). For instance, tumor necrosis factor-α (TNF-α), interleukin-1 (IL-1), interleukin-6 (IL-6) could interfere with the expression of many genes related to thyroid hormone metabolism and inhibit T3 production (37). Fourth, we found a higher incidence of ventricular arrhythmias in LT3S patients than in patients with normal FT3 level. FM patients often presented with various arrhythmias due to myocardial edema and electrocardiographic instability (38). LT3S can aggravate the left ventricular systolic dysfunction, which precipitates a higher incidence of ventricular arrhythmias. Collectively, these pathophysiological changes may eventually contribute to the poor prognosis of FM patients.

FM patients have a very poor short-term prognosis (6). A multi-center study suggested the early application of a life support-based comprehensive treatment regimen, including MCS, respiratory support, immune modulation with sufficient doses of glucocorticoids and immunoglobulins, and antiviral therapy significantly, could significantly reduce in-hospital mortality in patients with FM (19). Therefore, early risk stratification is of great clinical importance and can guide clinical treatment for FM patients. Previous studies indicated that many risk factors, such as renal dysfunction, impaired cardiac function, prolonged QRS duration and QTc interval, were associated with poor in hospital and long-term prognosis in patients with fulminant myocarditis (39–41). In our study, LT3S was an independent predictor for poor prognosis of FM patients. Consequently, FT3 should be considered to be a biomarker for risk stratification of FM patients. However, whether LT3S patients should receive treatment remains controversial. Kyle et al. reported that long-term T3 replacement therapy could improve ejection fraction and contractile performance in a rat model of post-MI (42). Some clinical studies further confirmed the role of thyroid hormone replacement therapy in enhancing cardiac function in LT3S patients (43, 44). While most studies reported encouraging findings, data about effects of thyroid hormone replacement therapy on long-term prognosis has not been available yet. Additionally, the type of thyroid hormones supplementation, administration route, treatment dosage and duration varied among different studies. Therefore, it is very difficult to compare results among these studies. Moreover, the association between thyroid hormone replacement therapy and long-term clinical outcomes remains to be investigated in large randomized controlled trials.

This study had several limitations. First, this is a single-center retrospective study. Therefore, we were unable to conclude the causal relationship between LT3S and poor prognosis of FM. Second, the impact of LT3S on long-term prognosis of FM patients could not be assessed due to the absence of long-term follow-up. Third, FM was diagnosed mainly by clinical presentation, laboratory data, and imaging results, whereas endomyocardial biopsy was not routinely performed to confirm the diagnosis.

## 5 Conclusions

LT3S was an independent predictor of 30-day mortality in FM patients. Additionally, FT3 level was a strong predictor of 30-day mortality and may serve as an useful risk-stratification biomarker for FM patients.

## Data availability statement

The original contributions presented in the study are included in the article/supplementary material, further inquiries can be directed to the corresponding author.

## Ethics statement

The studies involving human participants were reviewed and approved by the ethics committee of the First Affiliated Hospital of Zhengzhou University. The patients/participants provided their written informed consent to participate in this study.

## Author contributions

GM and XZ contributed to the conception and design of the study. GM, SP, YZ, MD, and LB collected the clinical information. GM wrote the first draft of the manuscript. SP and XZ reviewed and revised the manuscript. All authors contributed to the article and approved the submitted version.
